# Doping in Racing Pigeons (*Columba livia domestica*): A Review and Actual Situation in Belgium, a Leading Country in This Field

**DOI:** 10.3390/vetsci9020042

**Published:** 2022-01-22

**Authors:** Didier Marlier

**Affiliations:** Bird, Rabbit and Rodent Clinic, Faculty of Veterinary Medicine, University of Liege, B4000 Liege, Belgium; dmarlier@uliege.be; Tel.: +32-4-366-40-13

**Keywords:** pigeons racing, doping control, Belgium, short review, corticoids, NSAIDs

## Abstract

Pigeon racing is a sport in which trained homing pigeons (*Columba livia domestica*) are released between 60 and 1200 km from their loft and then have to return home as quickly as possible. The first race was held in 1818 in Belgium and since then, Belgium has led the world in pigeon breeding. Unfortunately, as in other sports, doping has become a major issue and doping controls have been implemented. This review provides information about pigeon racing, rules from the Royal Federation Colombophile of Belgium, and laws applicable in Belgium as doping control issues cannot be understood without including them as part of pigeon racing. The main pharmacological data concerning corticoids, non-steroidal anti-inflammatory drugs, anabolic steroids, pain relievers and narcotic analgesics, bronchodilators and β-agonists, drugs acting on the central nervous system and other performance-enhancing drugs, in addition to methods relevant to doping in pigeons are presented. Moreover, the chosen matrix and analytical methods are described.

## 1. Introduction

It is impossible to determine when humans invented “sport”, that is, when physical activities left the realm of simple concerns of “struggle for life” and became activities of leisure. However, regardless of when the concept of sport was invented, the use of substances to increase performance was known to Chinese physicians who recommended the use of Ma Huang (an extract from the plant genus *Ephedra*) to enhance performance over 5000 years ago [1]. Thus, although the word “doping” only appeared in 1889 in the English dictionary [2], the concept of doping (i.e., the use of methods to enhance athletic performance) precedes modern concepts of sport and this might well be the case in pigeon racing.

Substances and methods used in racing pigeons have changed along the years. In the 1970s, empirical usage of high vitamin doses, food supplements, coffee beans and even strychnine or arsenic (Fowler liqueur) were commonly reported (Vindevogel, personal communication) [3]. In the 1990s, the use of corticosteroids and anabolic steroids appeared and quickly emerged as a key issue in pigeon sports due to animal welfare concerns [3]. This resulted in the publication of a Royal Decree (14 February 1995) identifying the prohibited performance-enhancing substances in pigeons [4] and establishing punitive measures for transgressors of the decree [4]. Shortly after, the first internal regulations for combating doping in pigeons were implemented in accordance with the Royal Federation Colombophile of Belgium (RFCB/KBDB) [5].

The control of doping in racing pigeons presents an extremely difficult challenge when compared with human or equine competitive sports. Indeed, competitions may last several days from the moment pigeons leave their loft until the time they return home. The choice of samples for doping control presents another problem because in birds, droppings consist of both feces and urine. Additionally, the lack of pharmacological studies dedicated to pigeons provides another complicating factor.

This paper intends to provide basic information about pigeon racing as doping control issues in pigeons cannot be understood without first learning about the sport of pigeon racing. Moreover, it provides an overview of all aspects of doping control in racing pigeons based on the Belgian situation, as Belgium may well be the world’s leading country in this field to date.

### 1.1. The Wild, Domestic and Racing Pigeons (Columba livia)

The domestic pigeon (*Columba livia domestica*) is one of 344 species of pigeons and doves in the family columbidae, order columbiforme [6] and is derived from its wild ancestor the Rock Dove (*Columba livia*) originating from the rocky areas of Africa and Eurasia. Despite considerable uncertainty concerning the chronological and geographic origins of the domestic pigeon, they might indeed be the first domesticated birds and one of the earliest domesticated animals, with an initial domestication that may have taken place 5000 years ago in the Mediterranean region [7,8,9]. Food availability for humans was a likely starting point of domestication but pigeons were soon selected for exhibition and for their homing ability. Today, there is a large number of domestic breeds among them being the racing pigeon breed. The racing pigeon breed was selected by the crossing of numerous other breeds (Smerle, French Cumulet, English Carrier, …), resulting in birds with a high endurance, an ability to fly for hours and an extraordinary ability to navigate back to their homes over great distances [10].

Their highly developed orientation abilities based on primitive rock pigeon feeding and nesting behaviors were quickly recognized. Rock pigeons build their nests atop rock ledges in holes in the cliff and fly up to 20–30 km inland to find food and then must navigate their way back to their nests. The use of pigeons as messengers was reported during the accession to the throne of Ramses III around 1198 BC. Since Roman times up until World War II they were used as messengers during conflicts [3]. During the Middle Ages pigeon breeding was among one of the nobility and clergy privileges. This practice halted at the end of the French Revolution when privileges were abolished, which subsequently led commoners to keep pigeons either for food, exhibition or competitions [3]. The first race was held in 1818 in Belgium over a distance of about 160 km. Since then, pigeon racing evolved into a popular sport in other European and non-European countries such as France, Germany, the Netherlands and the United States [3]. Today, pigeon racing has virtually become a worldwide activity. At an international level it is supervised by the International Colombophile Federation (FCI) that was established in 1937 in Brussels [11]. At the moment of writing, there are 68 national federations that are members of the FCI with 11 national federations applying for membership [12].

For convenience, the term “pigeon” will be used for the rest of this article with reference to the “homing pigeon”.

### 1.2. Pigeon Racing

For pigeon racing, trained pigeons are driven by their fanciers (a pigeon fancier is a person who keeps and breeds pigeons) to their respective local federation where they are placed in transport baskets (basketing). Then, the transport baskets from different local federations are grouped and transported by road in specially equipped trucks to the releasing place. At a specific time and location, all the baskets are opened together and pigeons must fly back to their home lofts located between 100 km and over 1000 km from the starting place. In Belgium, the distance categories are sprint (around 100–150 km), middle-distance (around 250–500 km), long-distance (around 700–900 km) and very long-distance (around 1000–1200 km) [3,5,13,14,15]. Moreover, the competitions are organized according to age categories with classifications of ”young” (pigeons born in the year), “yearling” (pigeons of the previous year) and “old” (pigeons older than one year). For the yearlings and the old, a distinction is made between male and female for classification. Pigeons usually start racing at about 6 months of age and can still be in competition at over ten years old.

The competition results are established on the basis of the average speed achieved between the place of release and the home loft. Indeed, each racing pigeon has a specific numbered tamper-proofed metallic ring that is set in place around 1 week of age. It also contains an electronic tiny chip with a number linked to the official matricula number of the metallic ring. The time of release is known and the time of arrival is automatically and electronically recorded by specific antenna linked with tamper-proof data-takers that are set at the entrance of the home loft. The precise loft geographical coordinates are recorded, enabling the average speed to be calculated. One of the main advantages of competition day is a type of freedom for fanciers, who are not required to remain in their lofts until the return of their pigeons [3,5,13,14,15].

Pigeon trade accompanies the sport of pigeon racing. Belgium has long been known as “the place to be” as far as pigeon trade is concerned. Indeed, based on their spectacular results in races and concomitant century-long genetic selection, Belgian homing pigeons and pigeon breeders are considered as the best on earth [16,17,18,19,20]. Therefore, the homing pigeon trade is both extremely active and lucrative in Belgium. These last years, it has almost exploded with the arrival of Asiatic people, mostly Chinese fanciers [16,21,22]. At the time of writing, the record price per pigeon are EUR 1,252,000 in 2019 and EUR 1,600,000 in 2020 in Belgium, however, in 2018 a pigeon sold for EUR 2,780,000 in China [17,18,20,22]. Today, there are even internet platforms specialized in the pigeon trade with the highest revenue from the sale of birds ever held in pigeon business being EUR 9,551,200 in 2020 [23].

## 2. Doping Control

### 2.1. Rules and Laws

In Belgium, protection of pigeons during competition is regulated by rules from the KBDB-RFCB (Royal Federation Colombophile of Belgium) [5,24,25] and laws from the Brussels-Capital, Flemish or Walloon governments. Indeed, animal welfare has been one of the first regionalized competences since 1 July 2013. At the time of writing, laws are about the same in the three regions. Taking Wallonia as an example, specific laws are directly or indirectly dedicated to pigeon racing within the strict framework of animal welfare [4]. The most important ones are the laws of erected on 14 February 1995 establishing a list of prohibited products improving performance in pigeons and the laws instituted on 23 September 1998 on the protection of animals during competitions [4]. On the other hand, the KBDB-RFCB rules [5,24,25] are dedicated to the repression of sports fraud including the use of doping substances in the context of competitions. For completeness, if racing pigeons enter the food chain, a rare occurrence in Belgium but can happen on occasion, certain specific regulations from the Federal Agency for the Safety of the Food Chain (FASFC) must then be followed for possible residues in the meat [26].

An in-depth description of these laws and rules remains outside the scope of this review. Briefly, only healthy pigeons can participate in competitions that must be agreed upon by the local authorities. Competition requirements may not exceed the physiological capacities of the animals involved although these capacities are not defined by law. The Minister of Animal Welfare is responsible for laying down the necessary and legal conditions to be respected by the organizers of pigeon races with regard to the transport of pigeons by road, the collective release of pigeons taking place on Walloon territory, and the control of the use of prohibited substances fixed by the Royal Decree (14 February 1995). It also stipulates the list of prohibited products improving performance in pigeons i.e., corticosteroids; β-agonists; anabolic steroids; non-steroidal anti-inflammatory drugs and substances that impede the detection of the four products mentioned above [4]. The Minister of Animal Welfare may also lay down the conditions for legitimizing the persons responsible for detecting the use of these substances [27].

The KBDB-RFCB rules [5,25] which take precedence over FCI rules [28] precisely stipulate what should be considered as ”doping practices” and how they will be sanctioned. Moreover, they stipulate how and what type of samples may be taken. Refusal and/or the inability of taking a sample by the owner or their employee will also be considered a violation of doping control rules and are consequently punished in accordance with these rules. The list of banned substances is broader and more precisely defined in the KBDB-RFCB rules than in the laws of 1995 [4] and include corticosteroids, bronchodilators (including β-agonists), anabolic steroids, nonsteroidal anti-inflammatory drugs, narcotics, analgesics, substances that influence the nervous system (including caffeine), synthetic hormones, growth stimulators and mucolytics. This list of prohibited substances is given for informational purposes only and is detailed in a particular ”Red list” document [25] to better inform pigeon owners [5,25]. It must be emphasized that a distinction is clearly made between non-endogenous and endogenous substances, and between substances that might never be found in the body of pigeons as a result of dietary contamination and those that could be linked with consumption of contaminated food. Exogenous substances must not be present in doping samples regardless of the concentration. Endogenous substances and substances which may occur naturally in food (for example as a food contaminant) are only prohibited if they are detected in doping samples in amounts indicative of artificial administration [5].

The choice of pigeons that should be controlled is either selected at random or is based on the observation of particularly impressive results. Regional federations actively monitor competition results and point out those that might look abnormal. They inform the head of the national antidoping controller who then contacts the team of doping controllers that must travel to the suspected loft to perform sampling. The owner is fully responsible for any administration of substances to their pigeons. The competent bodies of the KBDB-RFCB are authorized to conduct the collection of droppings and/or feathers and/or blood of carrier pigeons and its members, at any time and in any location, in order to search for the presence of prohibited substances. Therefore, for all homing pigeon competitions, all ranked pigeons must remain in their fancier’s loft for control by the KBDB-RFCB or by the organizer, for a minimum of 5 working days from the end of the competition [5]. Samples may also be taken from the drinking water within the loft. Sampling is carried out by the competent persons mandated by the RFCB. Samples may be taken in the absence of the owner who must indicate the details of a representative contact person [5].

Currently, most of the anti-doping controls are carried out after the competitions. However, the possibility of carrying out specific targeted controls before competitions, i.e., at basketing of pigeons is being strongly considered.

### 2.2. Sample Collection

There has been some debate over which samples should be taken for doping control in pigeons. According to the KBDB-RFCB rules, the competent KBDB-RFCB authorities can obtain samples of feces, feathers, blood and drinking water of their members’ racing pigeons at any time and location in Belgium [5,25] but only feces and drinking water are virtually sampled.

Feces are the most commonly chosen matrix. They are easily collected either from specific animals placed in baskets or more generally from the loft. Specific methods were developed for the detection and quantification of doping compounds in fecal samples from racing pigeons [29,30,31].

Blood sampling is exceedingly stressful for pigeons. It seems overly risky for owners and can lead to discussions and reproaches if the sampled pigeons underperform after sampling. Nevertheless, blood sampling falls within the professionally recognized scope of veterinary practice and can only be performed by a licensed veterinarian. Moreover, the maximum concentration of a substance in blood often quickly decreases and falls below the limit of detection. This is critical in pigeon racing as the delay between basketing and the return to loft can reach several days.

Feathers were proposed as a likely interesting biological matrix for doping control in pigeons as the time duration for the detection of drugs would be longer in feathers than in other matrixes. Furthermore, feathers would always be available and easily collected. A methodology for the simultaneous detection of clenbuterol, prednisolone, betamethasone and budesonide was developed but the proof of applicability of this method in real samples was only verified for betamethasone [32]. Unfortunately, although potentially promising, this method was not applicable in the field. Indeed, once they have finished growing, feathers are essentially dead structures and for a time-point doping control during the flight season, only mature contour feathers and, among them, only covert feathers (tectrices) can be sampled. Clearly, neither flight (remiges) nor tail (rectrices) feathers can be plucked without impairing the ability to fly. However, in pigeons whereas the small feathers (semiplume, down, filoplume and bristle) molt almost year-round, the molt of contour feathers usually starts when the third primary remiges have molted and reaches its peak when the fifth primary remiges have molted [3]. Thus, in adult pigeons the contour feathers molt between August to mid-November and drugs can accumulate in feathers only at this time. Despite some slight differences, molting of tectrices occur in the same time course in young pigeons. Thus, the putative advantage that the detection of drugs would be longer with feathers is also the main disadvantage. After feathers are grinded for analysis (sample preparation), it cannot be determined when putative doping agents might have been administered. Therefore, it would be very easy for cheaters to claim that drugs were given when pigeons were not participating in competition, as agreed in the rules. This already poses a problem for exogenous substances (those that can never be found in pigeons) but becomes further detrimental to the detection of non-endogenous substances and to those that may come from the consumption of contaminated feed that could accumulate throughout the feather growth period and could lead to false-positive tests. This could be the subject of very lengthy discussions with owners of pigeons found positive during doping control. The possible cross-contamination by sweat, fecal matter, or the environment must also be considered [33].

Oral fluids (saliva and/or crop samples) are a matrix that might be considered in the future. The sampling is minimally invasive and with a simple swab of saliva from the oral cavity and/or the content of the crop may be collected [34]. Oral fluids bear the same advantages and limitations of use in pigeons as in humans. Another question arises in pigeons. Considering the small size of the pigeon, the possibility of collecting sufficient material for doping control should be considered. To our knowledge, this matrix has never been the subject of published studies and is not currently used for doping control in pigeons.

In birds, droppings consist of both feces and urine. Although the possibility of isolating urine from feces by cloacal cannulation was demonstrated [35], the method was not simple enough to be implemented in doping control. Furthermore, as for blood sampling, cloacal cannulation falls within the professionally recognized scope of veterinary practice and can only be performed by a licensed veterinarian. Consequently, urine is not a matrix of choice.

For the obtainment of samples, only sterile and controlled material is used in order to avoid any possibility of cross-contamination. When samples (at least 4 g per sample, i.e., at least 8 g in total) have been taken, they are mixed thoroughly and divided into “A” and “B” samples which are sent to a laboratory for analysis. Of course, there is a secure chain of custody ensuring that samples cannot be tampered. Samples are correctly identified and handled so that they cannot be contaminated or degraded and are sent to a laboratory. After sampling, the owner can indicate all drugs or treatments that have been administered to the pigeons and can indicate the contact details of the attending veterinarian who prescribed or provided these possible treatments. The “A” sample is analyzed and if a prohibited substance is found in the “A” sample the owner is immediately notified by the doping officer of the KBDB-RFCB [5]. At this stage, the owner can request a counter analysis on the “B” sample. They have ten working days to inform the doping officer by registered letter. If the sample “B” is negative, the procedure stops and the costs of analyzes are borne by the KBDB-RFCB. If the sample “B” is positive the doping officer of the KBDB-RFCB informs the owner by means of a registered letter [5]. The owner may then address a recommended letter to the doping officer of the KBDB-RFCB with a counter argument that is transmitted anonymously to the scientific advisory commission (SAC). Members of the SAC (at least 3 members) carry out a scientific evaluation that must always be announced unanimously and is then transmitted to the Board of the KBDB-RFCB by the doping officer. The SAC evaluation can be consulted by all parties involved (KBDB-RFCB—owners—lawyers…) and is for information purposes only. Penalties are defined in the KBDB-RFCB rules and any subsequent sanctions are taken by the National Board of Directors and Management of the KBDB-RFCB, not by the SAC. [5]. At the laboratory level, samples “A” and “B” are frozen and kept until the end of the regulation procedure.

The discussion (pros and cons) as to whether the “B” sample should be analyzed by the primary laboratory or by a different laboratory at the same, as practiced in human sports or horse racing, bearing in mind that there are fewer laboratories with the expertise and equipment to perform doping control in pigeons than in other species.

### 2.3. Laboratory Analysis

A review of analytical methodologies published from 1990 to 2019, for doping detection in animals was recently published [33]. As might be expected the number of published papers (four papers) is very limited when compared with those dedicated to horses (*Equus ferus caballus*), greyhounds (*Canis familiaris*) and even camels (*Camelus dromedarius*) [33].

Mass spectrometry coupled with gas or liquid chromatography is the technique of choice for the control of prohibited substances in human athletes as well as in production and sport animals [33,36,37,38,39,40,41,42,43,44,45,46,47,48]. An initial screening is carried out to identify the possible presence of prohibited compounds and then to quantify if necessary [37]. Following this, the presence of prohibited substances is confirmed [37]. The analysis includes the following steps that are common to all analyzes to be done. The compounds of interest are extracted from the samples usually with an organic solvent. This step may be preceded by enzymatic hydrolysis in order to release the conjugated forms of the molecules [36,37,38,39,40,41,42,43,44,45,46,47,48,49]. The extracts are then purified and concentrated most often by passing the sample through solid phase columns adapted according to the physicochemical characteristics of the molecules. The choice of columns depends on laboratory though it is usually based on published data [36,37,38,39,40,41,42,43,44,45,46,47,48,49]. This purification step can also be carried out by evaporation of the solvent. Extracted samples are then analyzed [36,37,38,39,40,41,42,43,44,45,46,47,48,49]. Currently, because of their high separation power and their ability to analyze a large number of molecules in a single assay, High Performance Liquid Chromatography and Ultra-High-Performance Liquid Chromatography are the techniques of choice [36,37,38,39,40,41,42,43,44,45,46,47,48,49]. It is common to test more than 100 compounds from different therapeutic classes in a single run [37,38,39] [27]. For the identification of molecules, tandem mass spectrometry, also known as MS/MS or MS^2^, is currently used [37,38,39]. In this analytical method, two or more mass analyzers are coupled using an additional reaction step to increase their ability to analyze chemical samples. As a whole, this analytical approach allows a quick and unambiguous identification of specific drugs and afford the detection of substance levels from nanograms per milliliter (a part per billion) to picograms per milliliter (a part per trillion).

Contrary to what has taken place in horseracing [47], no international threshold (i.e., the drug concentration in a sample at which this sample is considered as positive for doping control) has been established to control the abuse of substances which might naturally occur in pigeons (non-endogenous or coming from the consumption of contaminated feed). Thus, the establishment of a threshold is the responsibility of each national federation. In Belgium, thresholds have been set for certain drugs by the SAC. This was performed by statistical analysis of indubitably negative samples. So far, these thresholds are confidential.

As for horse racing, laboratory accreditation plays a vital role in prohibited substance testing [47]. The World Antidoping Agency and the International Equestrian Federation have an accreditation system based upon compliance with two international standards the “ISO/IEC 17025” and the “International standards for Laboratories” [50,51,52,53]. The laboratory drug screening and confirmatory procedures for equine samples are accredited to ISO/IEC 17025 by an internationally recognized accreditation body, and operate within the current versions of both ISO/IEC 17025 and the ILAC-G7 document. Any proficiency testing organized by the FEI would comply with ISO/IEC 17043 [50]. The ISO/IEC 17025 is an international standard which specifies general requirements for the competence of testing and calibration laboratories [54]. As with horse racing, there are no standard methods for the analyses of pigeon doping control samples; therefore, the range of tests and the prohibited substances covered vary immensely from laboratory to laboratory [47]. In any case, the laboratories accredited by the KBDB-RFCB must demonstrate impressive skills and competence in the matter. This is based on an accreditation to ISO/IEC 17025 by a Belgian accreditation organization (BELAC) for analysis of tissues and fluids of animal origin. Laboratories must additionally contain all the equipment, techniques and competent personnel necessary for carrying out trace analyzes. Currently, there are only two accredited laboratories in Belgium [24], of which only one (CER Groupe-Marloie) performs the vast majority of analyzes.

Unfortunately, the sample detection time (i.e., the amount of time a substance can be detected in a biological sample collected after administration of a specific dosage, route and drug formulation) of most, if not all, substances are usually unknown in pigeons. In the Belgium laboratory, negative results (no detection of banned substances) are defined as lower than the detection capability (CC_β_). Non-endogenous prohibited substances positive tests are reported as greater than the decisive limit (CC_α_) that is indicated in µg/kg of matrix. All other prohibited substances e.g., those that might come from consumption of contaminated food products are quantified and reported in µg/kg of matrix.

### 2.4. Current Issues and Future

There are only a limited number of pharmacological studies performed on pigeons and most drug doses and dosages in this species are either extrapolated from other bird species or even only empirical. This applies both to the treatment of sick pigeons and to drug abuse in a doping context as it does not afford the definition of withdrawal time (i.e., the time before racing after a treatment was given to a pigeon to avoid a positive doping control taking inter-pigeon and inter-treatment variability into account). This raises the question of medication versus doping control. Moreover, if for some drugs there is a good allometric correlation of pharmacokinetic parameters (half-life i.e., the time taken for the plasma concentration of a drug to be reduced to half of its original value) between species, for many other drugs, mainly low hepatic extraction drugs, there is no allometric correlation [55,56,57,58]. Pharmacokinetic processes of drugs may significantly differ between birds and mammals and pharmacokinetic data on mammals can hardly be extrapolated to birds as such. Differences in pharmacokinetic behavior between bird species may also happen [56]. Thus, selection of an appropriate posology for birds has to be based on pharmacokinetic data of that specific bird species [56].

Contrary to human sport or horse racing, retrospective testing [47] has not yet been implemented. It should be considered in the coming years mostly to prevent an increase in the capacity gap i.e., the delay between the emergence of a new prohibited substance and a test developed for its identification in biological samples [47]. From this perspective, the feasibility (effect on substances, presumed degradation, …) of long-term storage of fecal samples must be considered and thoroughly studied. Relevant rules to allow retrospective testing and sanctions should also be implemented in the KBDB-RFCB doping regulation.

## 3. Doping Agents

This chapter does not intend to give detailed pharmacological data on the drugs presented and only data relevant to the doping of pigeons will be presented. To the best of the author’s knowledge and despite extensive research in the peer-reviewed scientific and grey literature, there is no published research study dedicated to the improvement of racing performances in pigeons following administration of specific drugs (e.g., double-blind comparison of flight performances of “doped” and control pigeons in a standardized flight test). On the contrary, field observations and confidential comments from pigeon fanciers and veterinarians clearly indicate that some substances have obvious effects on sports performance. In the absence of an official investigation carried out on a large scale by competent authorities and officially published, it is exceedingly difficult to know precisely the drugs and methods used in doping. Table 1 presents the estimated frequency of use of substances potentially administered by pigeon fanciers with the aim of influencing the sporting performances of carrier pigeons based on confidential field reports, or direct observations (D. Marlier) or unpublished confidential results of a survey conducted in 2017 in Belgium [59]. This table should be taken with caution and is only indicative as it is not based on published and peer reviewed data.

### 3.1. Corticoids

Contrary to mammals, corticosterone as opposed to cortisol is the main natural corticosteroid in birds although an extremely limited amount of cortisol is produced [60]. There are only some limited data available on the effects of physical activity on corticosterone levels in pigeons. However, only a slightly increased corticosterone level was observed in homing pigeons after flights of more than 180 km [61] and only a slightly elevated corticosterone concentration, well below the levels after stimulation with adrenocorticotropic hormone (ACTH) [62] was reported. In pigeons the cortisol/corticosterone ratio varies between 0.07 and 0.3 [63]. This is important in the framework of doping control because only cortisol may be transformed into prednisolone by microbiological dehydrogenation [64] and consequently when prednisolone is found in pigeon feces, it almost certainly corroborates an exogenous administration. There is no corticosterone product on the market at the time of writing.

Synthetic corticosteroids are the most frequently studied substances in pigeons. Glucocorticoids, generally administered in the form of eye drops or in drinking water, are known to have been used as doping agents in pigeon sports [30,60,65,66,67] for a long time and might well remain in use today, especially in the form of drops for human use that pigeon fanciers obtain either abroad (e.g., Netherlands, Eastern Europe) or via personal medical prescriptions.

One of the main effects of glucocorticoid treatment for doping in pigeons is the inhibition of the natural molt of primary flight feathers. Thus, the young birds treated retain all their flight feathers and therefore have a higher aerodynamic efficiency than the animals with molted flight feathers, particularly if the last 4 primary flight feathers are involved. These young pigeons can compete longer during the competition season [3,65]. The effects of corticosteroids on molting have been studied in pigeons younger and older than 1 year of age [65]. Different doses, dosages, corticosteroid types and routes of administration have been tested. In birds less than 6 months old treated with eye drops 3 times per week for 9 weeks, the inhibitory action on molting is variable with prednisolone (0.5 mg) and complete for animals treated with dexamethasone (0.1 mg) and fluocinolone (0.01 mg). Lymphoid depletion of the Fabricius bursa and spleen has been observed. Prednisolone, administered to the eyes, was less toxic (lower treatment-related weight loss, upper hemoglobinemia and lower serum creatinine close to the values observed in control animals) than dexamethasone and fluocinolone acetate. The effects observed following the use of eye drops appear to be reversible in young pigeons [65].

In adult pigeons, the toxicity of triamcinolone administered at doses of 0.125, 0.250, 0.5 and 1 mg intramuscularly (IM) once a week for 6 weeks was proportional to the dose injected. Muscle atrophy and vacuolation of the pectoral muscles, fatty degeneration of the liver and kidneys, and atrophy of the genitals were observed. Calcemia and triglyceridemia were significantly increased compared to control animals [65]. The type of glucocorticoid used had a significant effect on hemoglobin, blood sugar, and creatinine. Corticoids induced immunosuppression resulting in respiratory infection with *Aspergillus fumigatus* that lead to death of one pigeon [65] while aspergillosis is considered as extremely rare in non-immunocompromised pigeons. This immunosuppressive effect of corticoids in pigeons was already observed in a previous study [67] and confirmed later [66]. Long-term corticosteroid treatment can also lead to impaired fertility, in both male and female pigeons, which can last up to 3 months after cessation of treatment [65].

In addition to their effects on molting, glucocorticoids increase blood triglycerides that are an easily usable energy supply for pigeons both during short and long distance flight [68]. Thus, the increase in triglyceridemia following glucocorticoid administration might participate in the doping effect. A cerebral effect (amphetamine-type euphoria) such as the one described in humans [69] and anti-inflammatory and analgesic effects [69] might also play a role, as molting effects alone cannot explain the reported performance improvements.

### 3.2. Non-Steroidal Anti-Inflammatory Drugs (NSAIDs)

NSAIDs are perhaps the most common class of analgesic drug prescribed in small animal medicine. Despite the lack of specific studies in birds, it is usually assumed that NSAIDs have a similar mechanism of action in both mammals and birds. Adverse effects of NSAIDs have not been fully substantiated in birds but they might be the same, with the most common reported adverse effect of NSAID usage in avian species being on renal tissue and function [70]. To date, no commercial formulations are available for use in birds in Belgium neither for pet birds nor for poultry and off-label use of these drugs in birds occurs frequently. The most common NSAIDs used in avian medicine include meloxicam, carprofen, ketoprofen, celecoxib and piroxicam [70]. In Belgium meloxicam, carprofen, ketoprofen are available as a registered veterinary medicine whereas celecoxib and piroxicam are restricted to human medicine. In the framework of doping, all NSAIDs for both veterinary and human use are likely to be employed by pigeon fanciers.

Salicylic acid is the active metabolite of acetylsalicylic acid (aspirin). Salicylates are natural chemicals produced by plants [71]. They are found in fruits and vegetables and help protect plants against disease and insects. Salicylic acid is naturally present in willow (genus *Salix*), meadowsweet (*Filipendula ulmaria*) and alfalfa (*Medicago sativa*). Extracts of certain plants, such as willow (genus *Salix*), myrtle (*Myrtus communis*), poplar (genus *Populus*) and meadowsweet (*Filipendula ulmaria*), which are rich in salicylates, have been used for millennia to treat human diseases [72]. Alfalfa is not a natural part of the pigeon diet but some food supplements and “herbal teas” frequently given to pigeons by fanciers contain variable levels of salicylic acid. The presence of salicylic acid during doping control could be linked with consumption of contaminated food. To avoid false-positive doping, a control threshold (for confidential use in KBDB-RFCB) was established for salicylic acid. In Belgium, there are registered veterinary drugs containing salicylate but none are registered for pet birds or poultry.

NSAIDs pharmacokinetic data strongly differ between mammals and birds and even between bird species [55,56,58]. The intravenous disposition of three (sodium salicylate, flunixin and meloxicam) commonly used NSAIDs in veterinary medicine, after single intravenous administration was studied in broiler chickens (*Gallus gallus*), ostriches (*Struthio camelus*), ducks (*Anas platyrhynchos*), turkeys (*Meleagris gallopavo*) and pigeons. Striking differences were observed among the species with some interesting differences, particularly in pigeons. The half-life of meloxicam (2.4 h) and salicylic acid (15 h) in pigeons was around three- to five-fold longer than in the other bird species [58].

The metabolic excretion pattern of sodium salicylate and its metabolites (gentisic acid, salicyluric acid and double conjugated ornithine metabolite) were studied in broiler chickens (*Gallus gallus*) and homing pigeons [73]. Salicylic acid, gentisic acid and salicyluric acid were found in pigeon droppings but not conjugated with ornithine. Contrary to chickens that form ornithine conjugates with simple carboxylic acid drugs, pigeons form glycine conjugates. A slower elimination pattern for salicylates and a longer excretion time for salicyluric acid in pigeon droppings was observed. In pigeons, the main site of glycine conjugation may be the kidneys. This might explain the long plasma half-life of salicylic acid in pigeons if pigeon kidneys bear a reduced metabolic capacity for salicylate [73].

Diclofenac appears to be rapidly toxic in pigeons with a 20% mortality at a dose of 0.25 mg/kg for 7 days, and up to 60% mortality at 20 mg/kg for 7 days [74]. Pigeons administered 10 and 20 mg diclofenac/kg were diagnosed with visceral gout. The kidneys and liver were enlarged. Histologically, the kidneys showed acute renal necrosis and the livers revealed fatty changes and necrosis of hepatocytes. The kidneys also exhibited uric acid crystal aggregates and associated lesions in the parenchyma [74]. Meloxicam at a dose of 0.4 mg/kg SID for 5 days had significant anti-inflammatory effects in pigeons with only limited observed adverse secondary effects, although there is no guarantee that tolerance of meloxicam could occur in pigeons as no residual concentration or true toxicological studies have been performed in pigeons to date [75]. The analgesic effects of 2 doses of meloxicam on the degree of postoperative orthopedic pain in pigeons was assessed [76]. Administration of meloxicam at 2.0 mg of meloxicam/kg, IM, immediately after surgery and then 2.0 mg/kg, PO, every 12 h for 9 days provided quantifiable analgesia that appeared safe in experimental conditions [76].

The pathologic effects of carprofen at 2, 5, or 10 mg/kg IM once daily for 7 days was evaluated in a pigeon model [77]. No clinical signs developed after administration of carprofen in pigeons in this study, suggesting that IM carprofen may best be reserved for short term use in birds with acute injuries [77]. However, neither the anti-inflammatory effects nor the analgesic effects were evaluated in this study.

### 3.3. Anabolic Steroids

Anabolic steroids such as nandrolone laurate have been used as doping agents in pigeons (D. Marlier, personal observation) but neither their pharmacological effects on pigeons nor their enhancing performance abilities have been studied to date. Anabolic steroids might increase the muscle mass of pigeons and could reduce the recovery times after competitions as observed in other species [78,79].

Based on testosterone, boldenone, and nandrolone are natural steroids that can be detected in pigeon droppings. As with other species, the concentrations may vary depending on the gender. For these substances, it is therefore important to establish thresholds that account for gender. This has not been undertaken to date.

The excretion of boldenone by racing pigeons has been studied. Two groups of 4 female pigeons aged 6 to 12 months received a single IM dose of boldenone at a dose of 1 mg/kg or 10 mg/kg. The presence of boldenone in feces was detected by ELISA (measurement of total immunoreactivity) and confirmed by HPLC/ELISA for up to 31 and 49 days in groups 1 and 2, respectively [31,80].

### 3.4. Pain Relievers and Narcotic Analgesics

Pain can be defined as a distressing sensation as well as an emotional experience linked to actual or potential tissue damage, with the sole purpose of notifying the body’s defense mechanisms to react towards a stimulus in order to avoid further tissue damage [81]. All vertebrate animals share similar neuroanatomic and neuropharmacological components required for nociception, detection, transmission, and response to noxious stimuli. Thus, pain exist in birds although it is difficult to define and recognize when birds sense pain furthermore when prey species such as pigeons are considered. Indeed, prey species often only demonstrate cryptic and subtle changes [70]. It is even more challenging to objectively determine whether a pain medication is effective in an avian patient [70] 

The efficacy of pain relievers and narcotic analgesics as doping agents would be linked to a reduction of muscle pain during flight. For opioids, some form of euphoria that results from stimulation of the brain’s reward system involving the neuronal µ receptors and dopamine [82], and certain effects on the endocrine system [83] might also play a role.

Paracetamol, also known as acetaminophen, is one of the most popular and widely used drugs for the treatment of pain and fever in humans [84]. Paracetamol has an analgesic effect in pigeons [85] without a demonstrated nephrotoxic potential, at least in chickens [86]. Paracetamol occupies a unique position among analgesic drugs, both for the type of pain relieved and the side effects. Paracetamol is ineffective as for intense pain. It is ineffective in pain arising from smooth muscle spasm in hollow viscera [84]. Paracetamol is entirely synthetic and is not known to occur naturally [87]. However, paracetamol may accumulate in aquatic environments through several emission sources from manufacturing facilities, consumer use and disposal, and hospital waste [88] and have been detected in surface waters, wastewater, and drinking water throughout the world [88], although at very low concentrations in drinking water [89,90] that appears unable to interfere with the results of an anti-doping control. Paracetamol abuse has already been found in pigeon racing.

The action of narcotic analgesics is based on binding to specific membrane receptors κ, δ and especially the µ receptors located in the central and peripheral nervous systems. In birds, opioid receptor distribution, density, and functionality are far less studied than in mammals [70]. The analgesic effects of opioids have a wide range of clinical efficacy depending on the avian species studied [91]. In birds, contrary to mammals, µ receptors are less strongly represented in the prosencephalon and mesencephalon than κ and δ receptors [70]. In pigeons, the regional distribution of µ, κ, and δ receptors in the forebrain and midbrain appear similar to mammals but the κ and δ receptors are more prominent in the pigeon forebrain and midbrain than µ receptors. In the pigeon forebrain 76% of opiate receptors appear to be of κ type [70].

In Belgium there are registered veterinary drugs containing buprenorphine, butorphanol, fentanyl, methadone, and tramadol. The abuse of these drugs as doping agents in pigeons is unknown to date but cannot be excluded.

Butorphanol is commonly used for avian analgesia but its use has never been fully studied in pigeons.

Buprenorphine at 0.25 mg/kg and 0.5 mg/kg IM in pigeons increases latency period for withdrawal from a noxious electrical stimulus of 2 and 5 h, respectively [70]

In 2016, a positive case of doping with morphine was detected by the KBDB-RFCB but was eventually considered as a case of food contamination [92,93] due to the consumption of poppy seeds (*Papaver somniferum*) that are regularly integrated into food supplements for pigeons, and known to contain varying concentrations of morphine, codeine, thebaine, noscapine, and papaverine [94,95]. The latex of the opium poppy (*Papaver somniferum*) contains up to 80 different alkaloids. Mature poppy seeds do not contain latex but can be contaminated with opium alkaloids [96]. Pigeons do not willingly consume poppy seeds and if given free choice of seeds, show a preference for larger seeds. However, to avoid false-positive doping controls, a threshold (for confidential use in KBDB-RFCB) was established for morphine.

### 3.5. Bronchodilators and β-Agonists

Bronchodilators remain the basic treatment for airway disorders such as asthma and chronic obstructive pulmonary disease in human [97,98] and veterinary medicine [99]. Their relatively short duration of action, even for long-acting bronchodilators [97,98], severely limits the value of their use in pigeon racing. Furthermore, the anatomy of the respiratory system differs greatly between birds and mammals and although bronchodilators are generally recommended for treatment of small airways diseases [100] there is no available publication clearly demonstrating their true efficacy.

On the contrary, in the framework of doping, the anabolic effects of beta-adrenoceptor agonists, their lipolysis effects and their effects on recovery time after an injury must be taken into account [33,78].

Clenbuterol abuse has been reported in pigeons and clenbuterol may be detected in feces up to one day after administration of a single dose (0.8 µg/kg, PO) [30]. The performance-enhancing effects of clenbuterol in pigeons remain unknown.

### 3.6. Drugs Acting on the Central Nervous System

Stimulant drugs might be used for doping in pigeons.

Due to its limitation of use (short-term effect and injection routes only) doxapram is of no interest for doping in pigeons.

Methylxanthines (caffeine, theobromine and theophylline) with their known stimulating effects on the cerebral cortex and spinal cord resulting in improved cognitive, physical and occupational performances can be potent doping agents [101,102]. The pharmacology, metabolism and mechanism of action of caffeine has recently been reviewed [102]. An increased muscle activity resulting in faster and more powerful muscle contractions was demonstrated [103]. Caffeine seems to be regularly administered to pigeons mainly in the form of tea that usually also contains other alkaloids such as theobromine and theophylline which are additionally considered as doping agents. As in humans, it can be assumed that low doses of caffeine do not present a problem. Conversely, large doses could adversely affect animal welfare. However, no studies have been performed in pigeons to date. A threshold (for confidential use in KBDB-RFCB) was established for caffeine.

Ephedrine and pseudoephedrine are natural substances derived from the Ephedra plant. Their ability to improve athletic performance is still debated [1,104]. On the contrary, amphetamines are synthetically prepared substances, with a structure and action similar to that of ephedrine. Both drugs often make the user feel more alert and energetic. Amphetamines suppress fatigue and may lead to improved physical performance though this effect is still debated [105,106,107].

One amphetamine positive case was detected in 2016. One atropine positive case was considered to be the result of food contamination and a threshold (for confidential use in KBDB-RFCB) was established for atropine.

### 3.7. Other Drugs and Methods 

While hematocrit and hemoglobin are key determinants of aerobic capacity and exercise performance in humans [108,109] this relationship has not been demonstrated in birds, and of course in pigeons, to date. In yellow-rumped warblers (*Setophaga coronata*) the relationship between hematocrit and exercise performance would depend on flight altitude [110]. At ground level, flight performances were slightly improved in birds with high hematocrit whereas at high altitudes (around 3000 m) flight performances were significantly improved in birds with low hematocrit. Whether these results apply to pigeons of which flight altitudes vary from ground level to 1800 m is unknown. The relationship between hematocrit and exercise performance may depend on bird species.

Birds, as with mammals, respond to blood loss and blood destruction by increasing erythropoietin production, which stimulates erythropoiesis [111], but unlike mammals birds do not have the ability to store erythrocytes in the spleen and they even decrease hematocrit during exercise as a result of hemodilution [110,112,113]. Regardless, mammalian erythropoietin has no effect on avian hematopoiesis [114]. Thus, recombinant human erythropoietin drugs registered on the market are not active in pigeons.

The lack of links between hematocrit and sports performance deprives any interest in physical methods such as blood transfusions which also impose specific constraints on birds [115].

Intramuscular injection of ACTH (50 µg or 125 µg) in healthy pigeons causes a ten- to hundred-fold increase over baseline corticosterone concentrations [116]. Therefore, ACTH could be used as a performance-enhancing drug in pigeons.

Since 2017, mucolytics were added to the list of prohibited substances in the KBDB-RFCB rules. Bromhexine is a registered veterinary drug in Belgium with a poultry indication. Ambroxol is a registered medicinal product for human use. Both drugs have reported use in pigeons.

Although they are not in the red list [25], the abuse and misuse of antibiotics are a main concern in pigeon racing. In Flanders, homing pigeons would be treated on average every ten days with an antibiotic during the flying season [117]. The situation in Wallonia is unknown but may roughly be the same. Moreover, antibiotics are mainly used for prophylactic and subclinical infections, to overcome periods of stress, or as an attempt to improve sport performance [117]. Contrary to recommended veterinary practices and legal obligations, most of these antibiotics are acquired illegally, that is, without a prescription or the supervision of a veterinarian [117]. This has led to high levels of antibiotic resistance even for critically important antimicrobials (Marlier) [117,118,119,120]. Based on recommendations for the use of antibiotics which limit their use to sick animals and on the legal basis which prohibits sick animals from participating in competitions [4], measures should be taken to control this situation.

## 4. Conclusions

In Belgium, doping controls to date are managed by the KBDB-RFCB which operates with utmost diligence to track down illegal drug use in pigeon racing. Fecal samples remain the most suitable matrix for doping controls in pigeons as they are easily collected and allow the detection of most doping agents. However, pharmacological studies should be specifically carried out in order to better understand the real effects of drugs on the performance of pigeons and to determine withdrawal and detection times. Indeed, a balance must be found between the obligation to eliminate cheaters and other purposes such as the need to treat sick or injured pigeons during the racing season. Therefore, determination of the concentration range classifiable as doping should be carried out at least for endogenous substances, and for those that might be incidentally found after consumption of contaminated food.

Corticoids, anabolic steroids, bronchodilators and β-agonists, and amphetamines should remain banned as they bear few, if any, beneficial properties in pigeon medicine at least during the racing season.

NSAIDs and pain relievers should never be administered at the owner’s discretion, which, notably, is unequivocally illegal in Belgium and should only be used under veterinary supervision. This implies a precise reporting of the administered substances (doses, time, pigeon ring number, …) and partly places the risk and burden of a positive control on the veterinarian, which is unreasonable given withdrawal times remain unknown.

Methylxanthines and salicylic acid can be found in teas given to pigeons as part of a daily care routine. The amount of methylxanthine and salicylic acid may greatly vary between teas but are far below the level found in commercial drug preparations. Regardless, avoidance of herbal teas can be recommended when the food composition and/or analysis are not clearly stated.

As with other sports, there may be a gap between the number of drugs available and the number detectable, however, great efforts are made to ensure the integrity of the sport remains as sound as reasonably possible, and that animal welfare is protected. This is not only extremely important in the context of anti-speciesism activist groups, of which some activists advocate the removal of animals from sport-use, but also in order to maintain a reliable selection of racing pigeons, and the national and international pigeon trade.

## Figures and Tables

**Table 1:** Estimated frequency of use of substances potentially administered by pigeon fanciers with the aim of influencing the sporting performances of carrier pigeons based on confidential field reports or direct observations (D. Marlier) or unpublished confidential results of a survey conducted in 2017 in Belgium [39]. This table should be taken with caution and is only indicative as it is not based on published and peer reviewed data.

Drugs	Estimated Frequency of Use			
	Frequent	Occasionally	Infrequent	Unknown
Corticosteroids				
Prednisolone	X			
Methylprednisolone	X			
Dexamethasone		X		
Betamethasone				X
Fluocinolone				X
Triamcinolone				X
Non-steroidal anti-inflammatory drugs				
Meloxicam	X			
Diclofenac	X			
Salicylic acid	X			
Flunixin	X			
Other		X		
Anabolic steroids				
Boldenone		X	X	
Nandrolone				X
Testosterone				X
Pain relievers and narcotic analgesics				
Buprenorphine, butorphanol, others				X
Paracetamol		X		
Others				X
Bronchodilators and β-agonists				
Clenbuterol			X	
Others				X
Drugs acting on the central nervous system				
Doxapram				X
Caffeine (and methylxanthines)	X			
Amphetamines			X	
Other drugs and methods				X
ACTH			X	
EPO			X	X
GnRH			X	X
Bromhexine		X		
Ambroxol				X
Acetylcysteine				X
Antibiotics	X			

## Data Availability

Not applicable.

## References

[1] Abourashed E.A., El-Alfy A.T., Khan I.A. (2003). Ephedra in perspective—A current review. Phyther. Res..

[2] Müller R.K., Thieme D., Hemmersbach P. (2010). History of doping and doping control. Doping in Sports: Biochemical Principles, Effects and Analysis.

[3] Vindevogel H., Duchatel J.-P., Pastoret P.-P. (1998). Le Pigeon Voyageur.

[4] SPW Bien-Etre Animal Le Bien-Etre Animal en Wallonie. http://bienetreanimal.wallonie.be/home/legislation.html.

[5] KBDB-RFCB Les Règlements et Statuts. www.kbdb.be/fr/les-reglements-et-statuts/.

[6] Gill F., Donsker D., Rasmussen P. IOC World Bird List (v11.2). https://www.worldbirdnames.org/new/.

[7] Stringham S.A., Mulroy E.E., Xing J., Record D., Guernsey M.W., Aldenhoven J.T., Osborne E.J., Shapiro M.D. (2012). Divergence, convergence, and the ancestry of feral populations in the domestic rock pigeon. Curr. Biol..

[8] Driscoll C.A., Macdonald D.W., O’Brien S.J. (2009). From wild animals to domestic pets, an evolutionary view of domestication. Proc. Natl. Acad. Sci. USA.

[9] Gilbert M.T.P., Shapiro M.D., Smith C. (2014). Pigeons: Domestication. Encyclopedia of Global Archaeology.

[10] Levi W. (1977). The Pigeon.

[11] World Flexagon KFT FCI—Home. https://pigeonsfci.org/.

[12] World Flexagon KFT FCI Membership. https://pigeonsfci.org/membership/.

[13] KBDB-RFCB Lossingen in Beeld. https://www.kbdb.be/fr/lachers-video/.

[14] KBDB-RFCB Bureau D’Enlogements. https://www.kbdb.be/fr/bureaux-denlogement/.

[15] KBDB-RFCB Home. https://www.kbdb.be/fr/591-2/.

[16] Laurence J.C. Quand les Pigeons Valent des Millions. https://www.lapresse.ca/international/europe/2021-04-10/la-presse-en-belgique/quand-les-pigeons-valent-des-millions.php.

[17] Verbist A. Les Pigeons Belges S’envolent à Prix d’or Pour la Chine. https://www.lavenir.net/cnt/dmf20120303_00126177.

[18] François A. Le Pigeon le Plus Cher du Monde Vient de Flandre Occidentale. https://www.vrt.be/vrtnws/fr/2019/03/17/le-pigeon-le-plus-cher-au-monde-vient-de-flandre-occidentale/.

[19] Carbonaro J. The World’s Most Expensive Pigeon is a Belgian Racing Bird Worth $1.8m. https://newseu.cgtn.com/news/2020-11-16/The-world-s-most-expensive-pigeon-is-a-Belgian-racing-bird-worth-1-8m-VrxdRtr44w/index.html.

[20] Dupont G. Le Pigeon Voyageur le Plus Cher de L’histoire est Belge: New Kim Vendu 1 600 000 Euros!. https://www.lalibre.be/belgique/societe/2020/11/15/le-pigeon-voyageur-le-plus-cher-de-lhistoire-est-belge-new-kim-vendu-1-600-000-euros-EKRO2DSLZJGNRPQLOZAYWOEEJY/.

[21] Anonymous Belgique: Un Pigeon Vendu 1,6 Million D’Euros, un Record. https://www.20minutes.fr/monde/2908895-20201115-belgique-pigeon-voyageur-vendu-16-million-euro-record.

[22] Anonymous Jales Huang Wins Ace Pigeon Pioneer Club—Pigeon Sold for World Record of 2.78 Million Euros!. https://www.deduif.be/nl/nieuws/james-huang-wins-ace-pigeon-pioneer-club-pigeon-sold-world-record-278-million-euros.

[23] PIPA Trading BV PIPA Milestones. https://www.pipa.be/fr/pipa-milestones.

[24] KBDB-RFCB Liste des Laboratoires Reconnus. www.kbdb.be/fr/doping-labo-2/.

[25] KBDB-RFCB Liste Rouge. https://www.kbdb.be/images/RED%20LIST%202018%20Fran%C3%A7ais.pdf.

[26] AFSCA-FAVV Circulaire Relaitve à L’Introduction des Pigeons Dans la Chaîne Climentaire. www.favv-afsca.be/professionnels/productionanimale/animaux/circulaires/_documents/2015_06_15_FR_circ-ob_pigeons_v1.pdf.

[27] Vlaams Parlement Gemeenschaps-En Gewestregeringen Gouvernements de Communaute et de Region Gemeinschafts- und regionalregierungen. http://www4wvg.vlaanderen.be/wvg/ijh/vlaanderen/documentenregelgeving/20130913_Decemberreet_staatsblad.pdf.

[28] International Pigeon Racing Federation Anti-Doping Control. https://pigeonsfci.org/wp-content/uploads/2020/04/Anti-Doping-Control-EN.pdf.

[29] Moreira F.X., Silva R., André M.B., de Pinho P.G., Bastos M.L., Ruivo J., Ruivo P., Carmo H. (2018). Quantification of doping compounds in faecal samples from racing pigeons, by liquid chromatography-tandem mass spectrometry. J. Chromatogr. B Anal. Technol. Biomed. Life Sci..

[30] Hagedorn H., Zankl H., Grund C., Schulz R. (1996). Detection of doping compounds in the racing pigeon. Berl. Munch. Tierarztl. Wochenschr..

[31] Hagedorn H.W., Zankl H., Schulz R. (1997). Excretion of the anabolic steroid boldenone by racing pigeons. Am. J. Vet. Res..

[32] Moreira F.X., Carmo H., Melo A., André M.B., Silva R., Azevedo Z., Bastos M.L., De Pinho P.G. (2019). The use of feathers from racing pigeons for doping control purposes. J. Anal. Toxicol..

[33] Moreira F., Carmo H., Guedes de Pinho P., Bastos M.d.L. (2021). Doping detection in animals: A review of analytical methodologies published from 1990 to 2019. Drug Test. Anal..

[34] Huestis S.A.M.A. (2012). The potential role of oral fluid in antidoping testing. Nat. Med..

[35] Halsema W., Alberts H., de Bruijne J., Lumeij J. (1987). Collection and analysis of urine from racing pigeons (*Columba livia domestica*). Avian Pathol..

[36] Thevis M., Opfermann G., Schänzer W. (2003). Liquid chromatography/electrospray ionization tandem mass spectrometric screening and confirmation methods for β2-agonists in human or equine urine. J. Mass Spectrom..

[37] Peters R.J.B., Stolker A.A.M., Mol J.G.J., Lommen A., Lyris E., Angelis Y., Vonaparti A., Stamou M., Georgakopoulos C., Nielen M.W.F. (2010). Screening in veterinary drug analysis and sports doping control based on full-scan, accurate-mass spectrometry. TrAC Trends Anal. Chem..

[38] Badoud F., Grata E., Perrenoud L., Saugusty M., Rudaz S., Veuthey J.L. (2010). Fast analysis of doping agents in urine by ultra-high-pressure liquid chromatography-quadrupole time-of-flight mass spectrometry. II: Confirmatory analysis. J. Chromatogr. A.

[39] Badoud F., Grata E., Perrenoud L., Avois L., SAugusty M., Rudaz S., Veuthey J.L. (2009). Fast analysis of doping agents in urine by ultra-high-pressure liquid chromatography-quadrupole time-of-flight mass spectrometry. I: Screening analysis. J. Chromatogr. A.

[40] Judák P., Esposito S., Coppieters G., Van Eenoo P., Deventer K. (2021). Doping control analysis of small peptides: A Decemberade of progress. J. Chromatogr. B Anal. Technol. Biomed. Life Sci..

[41] Putz M., Piper T., Casilli A., Radler de Aquino Neto F., Pigozzo F., Thevis M. (2018). Development and validation of a multidimensional gas chromatography/combustion/isotope ratio mass spectrometry-based test method for analyzing urinary steroids in doping controls. Anal. Chim. Acta.

[42] De la Torre X., Iannone M., Botrè F. (2021). Improving the detection of anabolic steroid esters in human serum by LC–MS. J. Pharm. Biomed. Anal..

[43] OJanuaryperä I., Kolmonen M., Pelander A. (2012). Current use of high-resolution mass spectrometry in drug screening relevant to clinical and forensic toxicology and doping control. Anal. Bioanal. Chem..

[44] Salamin O., Nicoli R., Xu C., Boccard J., Rudaz S., Pitteloud N., Saugusty M., Kuuranne T. (2021). Steroid profiling by UHPLC-MS/MS in dried blood spots collected from healthy women with and without testosterone gel administration. J. Pharm. Biomed. Anal..

[45] Kioussi M.K., Lyris E.M., Angelis Y.S., Tsivou M., Koupparis M.A., Georgakopoulos C.G. (2013). A generic screening methodology for horse doping control by LC-TOF-MS, GC-HRMS and GC-MS. J. Chromatogr. B Anal. Technol. Biomed. Life Sci..

[46] Wong J.K.Y., Choi T.L.S., Kwok K.Y., Lei E.N.Y., Wan T.S.M. (2018). Doping control analysis of 121 prohibited substances in equine hair by liquid chromatography–tandem mass spectrometry. J. Pharm. Biomed. Anal..

[47] Wong J.K.Y., Wan T.S.M. (2014). Doping control analyses in horseracing: A clinician’s guide. Vet. J..

[48] Fromm M.F., Kim R.B., Thieme D., Hemmersbach P. (2010). Doping in Sports Biochemical Principles, Effects and Analysis.

[49] Olędzka I., Kowalski P., Plenis A., Bączek T. (2017). Evaluation of various approaches to the isolation of steroid hormones from urine samples prior to FASS-MEKC analysis. Electrophoresis.

[50] International Equestrian Federation FEI Standards for Laboratories. https://www.google.com.tw/url?sa=t&rct=j&q=&esrc=s&source=web&cd=&ved=2ahUKEwiJiJDp8sn1AhUnSGwGHbhiBskQFnoECAgQAQ&url=https%3A%2F%2Finside.fei.org%2Fsystem%2Ffiles%2FFEI%2520Standard%2520for%2520Laboratories%2520-%2520December%25202017_0.pdf&usg=AOvVaw3fE3Rxn84CAZPh38Xv_C9B.

[51] World Anti-Doping Agency Accreditation Process. www.wada-ama.org/en/what-we-do/science-medical/laboratories/accreditation-process.

[52] International Equestrian Federation Anti-Doping Rules. https://inside.fei.org/content/anti-doping-rules.

[53] World Anti-Doping Agency International Standard for Laboratories. https://www.wada-ama.org/en/resources/laboratories/international-standard-for-laboratories-isl.

[54] World Anti-Doping Agency Guidelines for Harmonization of Scopes of iISO/IEC 17025 Accreditation of WADA Anti-Doping Laboratroies. https://www.wada-ama.org/en/resources/science-medicine/guidelines-for-harmonization-of-scopes-of-isoiec-17025-accreditation-of.

[55] Goessens T., Antonissen G., Croubels S., De Backer P., Devreese M. (2016). Nonsteroidal anti-inflammatory drugs in birds: Pharmacokinetics, pharmacodynamics and toxicity. Vlaams Diergeneeskd. Tijdschr..

[56] Houben R., Antonissen G., Croubels S., De Backer P., Devreese M. (2016). Pharmacokinetics of drugs in avian species and the applications and limitations of dose extrapolation. Vlaams Diergeneeskd. Tijdschr..

[57] Huang Q., Gehring R., Tell L.A., Li M., Riviere J.E., Marchtin-Jimenez T., Sundlof S.F., Craigmill A.L. (1997). Interspecies allometric meta-analysis of the comparative pharmacokinetics of 44 drugs across veterinary and laboratory animal species. J. Vet. Pharmacol. Ther..

[58] Baert K., De Backer P. (2003). Comparative pharmacokinetics of three non-steroidal anti-inflammatory drugs in five bird species. Comp. Biochem. Physiol. C Toxicol. Pharmacol..

[59] Harmegnies N., Scippo M.L., Marchlier D. (2017). Rapport Final D’Activité “Contrôle de L’Utilisation de Substances Ayant Pour but D’Influencer les Prestations des Pigeons Lors de Compétition”—Projet Subventionné Par la Région Wallonne—DGARNE—Dossier D32-0294.

[60] Westerhof I., Van den Brom W.E., Mol T.A. (1996). Responsiveness of the glucocorticoid-suppressed pituitary-adrenocortical system of pigeons (*Columba livia domestica*) to stimulation with arginine vasopressin. American Association of Avian Pathologists Stable. Avian Dis..

[61] Haase E., Rees A., Harvey S.F. (1986). Flight stimulates adrenocortical activity in pigeons (*Columba livia*). Gen. Comp. Endocrinol..

[62] Rees A., Harvey S. (1987). Adrenocortical responses of pigeons (*Columba livia*) to treadwheel exercise. Gen. Comp. Endocrinol..

[63] Westerhof I. (1998). Pituitary-adrenocortical function and glucocorticoid administration in pigeons (*Columba livia domestica*). J. Avian Med. Surg..

[64] Kaul R., Mattiasson B. (1994). Biotransformation of hydrocortisone in prednisolone. Methods Enzymol..

[65] Duchatel J.P., Beduin J.M., Jauniaux T., Coignoul F., Vindevogel H. (1993). Premières observations sur l’utilisation des glucocorticoïdes comme agent dopant chez le pigeon voyageur. Ann. Med. Vet..

[66] Dhondt G., Ectors R., Mathys L., Ducatelle R. (1993). Inhibition of molting in pigeons by glucocorticoids. Vlaams Diergeneeskd. Tijdschr..

[67] Gröger U., Grimm F. (1989). Dexamethason und Prednisoloneinsatz bei Tauben [Dexamethasone and prednisolone use in pigeons]. Tierarztl. Prax..

[68] Schwilch R., Jenni L., Jenni-Eiermann S. (1996). Metabolic responses of homing pigeons to flight and subsequent recovery. J. Comp. Physiol. B.

[69] De Mondenard J.P. (2002). Cortisone et corticoïdes, l’euphorie des performances mais la certitudes des défaillances et des complications. Courr. Addict..

[70] Hawkins M.G., Paul-Murphy J. (2011). Avian analgesia. Vet. Clin. N. Am. Exot. Anim. Pract..

[71] Lefevere H., Bauters L., Gheysen G. (2020). Salicylic acid biosynthesis in plants. Front. Plant Sci..

[72] Duthie G.G., Wood A.D. (2011). Natural salicylates: Foods, functions and disease prevention. Food Funct..

[73] Baert K., Croubels S., Maes A., Hillaert U., Van Calenbergh S., De Backer P. (2004). Comparative metabolic excretion profile of sodium salicylate in broiler chickens and homing pigeons. J. Vet. Pharmacol. Ther..

[74] Hussain I., Zargham Khan M., Khan A., Javed I., Kashif Saleemi M. (2008). Toxicological effects of diclofenac in four avian species. Avian Pathol..

[75] Marchlier D., Jonckers F., Vindevogel H. (1997). Essai d’utilisation du méloxicam (Metacam^®^ Boehringer Ingelheim) chez le pigeon voyageur. Ann. Med. Vet..

[76] DesMarchchelier M., Troncy E., Fitzgerald G. (2012). Lair Analgesic effects of meloxicam administration on postoperative orthopedic ain in domestic pigeons (*Columba livia*). Am. J. Vet. Res..

[77] Zollinger T.J., Hoover J.P., Payton M.E., Schiller C.A. (2011). Clinicopathologic, gross necropsy, and histologic findings after intramuscular injection of carprofen in a pigeon (*Columba livia*) model. J. Avian Med. Surg..

[78] Lynch G.S., Schertzer J.D., Ryall J.G. (2008). Anabolic agents for improving muscle regeneration and function after injury. Clin. Exp. Pharmacol. Physiol..

[79] Veuthey J.-L. (2021). Chapitre 6: La traque aux molécules dopantes. La Chimie et le Sport.

[80] Habenicht U.F., Aitken R.J. (2010). Handbook of Experimental Pharmacology.

[81] Yam M.F., Loh Y.C., Tan C.S., Adam S.K., Manan N.A., Basir R. (2018). General pathways of pain sensation and the major neurotransmitters involved in pain regulation. Int. J. Mol. Sci..

[82] Benedetti F., Pollo A., Colloca L. (2007). Opioid-mediated placebo responses boost pain endurance and physical performance: Is it doping in sport competitions?. J. Neurosci..

[83] Vuong C., Van Uum S.H.M., O’Dell L.E., Lutfy K., Friedman T.C. (2010). The effects of opioids and opioid analogs on animal and human endocrine systems. Endocr. Rev..

[84] Bertolini A., Ferrari A., Ottani A., Guerzoni S., Tacchi R., Leone S. (2006). Paracetamol: New vistas of an old drug. CNS Drug Rev..

[85] Brune K.A.Y., Bucher K., Walz D. (1974). The avian microcrystal arthritis II. Central versus peripheral effects of sodium salicylate, acetaminophen and colchicine. Agents Actions.

[86] JayakuMarch K., Mohan K., Swamy H.D.N., Shridhar N.B., Bayer M.D. (2010). Study of nephrotoxic potential of acetaminophen in birds. Toxicol. Int..

[87] IARC Working Group on the Evaluation of Carcinogenic Risks to Humans (1990). Paracetamol. IARC Monographs on the Evaluation of Carcinogenic Risks to Humans.

[88] Wu S., Zhang L., Chen J. (2012). Paracetamol in the environment and its degradation by microorganisms. Appl. Microbiol. Biotechnol..

[89] Nott K., Gillet M., Carbonnelle P., Frippiat C., Moutier M., Ronkart S., Delloye F., Brahy V. Recherche de Substances Emergentes Dans les Eaux et Intéressant la Santé Publique et L’Environnement. Programme de Recherche IMHOTEP (Inventaire des Matières Hormonales et Organiques en Traces Dans les Eaux Patrimoniales et Potabilisables). https://www.google.com.tw/url?sa=t&rct=j&q=&esrc=s&source=web&cd=&ved=2ahUKEwiUwsq288n1AhXNsVYBHfHoBjAQFnoECAgQAQ&url=http%3A%2F%2Fetat.environnement.wallonie.be%2Ffiles%2FStudies%2F2018_IMHOTEP.pdf&usg=AOvVaw3AsaX6eoQmzZxtCcTmHnen.

[90] Al-Kaf A.G., Naji K.M., Abdullah Q., Edrees W.H.A. (2017). Occurrence of paracetamol in aquatic environments and transformation by microorganisms: A review. Chronicles Pharm. Sci..

[91] Fousse S.L., Golsen B.M., Sanchez-Migallon Guzman D., Paul-Murphy J.R., Stern J.A. (2020). Varying expression of mu and kappa opioid receptors in cockatiels (*Nymphicus hollandicus*) and domestic pigeons (*Columba livia domestica*). Front. Genet..

[92] Degreef M., Raats S., Op de Beek J., Covacci A., Eeens M., van Nuijs A., Maudens K. The pigeon poppy seed defence. Proceedings of the 56th TIAFT Conference, The International Association of Forensic Toxicologists.

[93] Raats S. (2017). De Farmacokinetiek van Opiaten na Toediening van Maanzaad bij Duiven. Ph.D. Thesis.

[94] Shetge S.A., Dzakovich M.P., Cooperstone J.L., Kleinmeier D., Redan B.W. (2020). Concentrations of the opium alkaloids morphine, codeine, and thebaine in poppy seeds are reduced after thermal and washing treatments but are not affected when Incorporated in a model baked product. J. Agric. Food Chem..

[95] Carlin M.G., Dean J.R., Ames J.M. (2020). Opium alkaloids in harvested and thermally processed poppy seeds. Front. Chem..

[96] Knutsen H.K., Alexander J., Barregård L., Bignami M., Brüschweiler B., Ceccatelli S., Cottrill B., Di Novemberi M., Edler L., Grasl-Kraupp B. (2018). Update of the scientific opinion on opium alkaloids in poppy seeds. EFSA J..

[97] Matera M.G., Page C.P., Calzetta L., Rogliani P., Cazzola M. (2020). Pharmacology and therapeutics of bronchodilators revisited. Pharmacol. Rev..

[98] Williams D.M., Rubin B.K. (2018). Clinical pharmacology of bronchodilator medications. Respir. Care.

[99] Montgomery J.B. (2019). Beyond steroids and bronchodilators—Investigating additional therapies for horses with severe equine asthma. Vet. Rec..

[100] Orosz S.E., Lichtenberger M. (2011). Avian respiratory distress: Etiology, diagnosis, and treatment. Vet. Clin. N. Am. Exot. Anim. Pract..

[101] Machnik M., Kaiser S., Koppe S., Kietzmann M., Schenk I., Düe M., Thevis M., Schänzer W., Toutain P.L. (2017). Control of methylxanthines in the competition horse: Pharmacokinetic/Pharmacodynamic studies on caffeine, theobromine and theophylline for the assessment of irrelevant concentrations. Drug Test. Anal..

[102] McLellan T.M., Caldwell J.A., Lieberman H.R. (2016). A review of caffeine’s effects on cognitive, physical and occupational performance. Neurosci. Biobehav. Rev..

[103] Cooper C.E.C., Beneke R., Jones G. (2008). Caffeine and other sympathomimetic stimulants: Modes of action and effects on sports performance. Essays Biochem..

[104] Powers M.E. (2001). Ephedra and its application to sport performance: Another concern for the athletic trainer?. J. Athl. Train..

[105] Roberts C.A., Jones A., Sumnall H., Gage S.H., Montgomery C. (2020). How effective are pharmaceuticals for cognitive enhancement in healthy adults? A series of meta-analyses of cognitive performance during administration of modafinil, methylphenidate and D-amphetamine. Eur. Neuropsychopharmacol..

[106] Dufka F., Galloway G., Baggott M., Mendelson J. (2009). The effects of inhaled L-methamphetamine on athletic performance while riding a stationary bike: A randomised placebo-controlled trial. Br. J. Sports Med..

[107] Sitte H.H., Freissmuth M. (2015). Amphetamines, new psychoactive drugs and the monoamine transporter cycle. Trends Pharmacol. Sci..

[108] Birkeland K.I., Stray-Gundersen J., Hemmersbach P., Hallen J., HAugust E., Bahr R. (2000). Effect of rhEPO administration on serum levels of sTfR and cycling performance. Med. Sci. Sports Exerc..

[109] Böning D., Maassen N., Pries A. (2011). The hematocrit paradox—How does blood doping really work?. Int. J. Sports Med..

[110] Yap K.N., Dick M.F., Guglielmo C.G., Williams T.D. (2018). Effects of experimental manipulation of hematocrit on avian flight performance in high- and low-altitude conditions. J. Exp. Biol..

[111] Campbell T.W. (2015). Exotic Animal Hematology and Cytology.

[112] Jenni L., Müller S., Spina F., Kvist A., Lindström Å. (2006). Effect of endurance flight on haematocrit in migrating birds. J. Ornithol..

[113] John J.L. (1994). The avian spleen: A neglected organ. Q. Rev.Biol..

[114] Yap K.N., Tsai O.H.I., Williams T.D. (2019). Haematological traits co-vary with migratory status, altitude and energy expenditure: A phylogenetic, comparative analysis. Sci. Rep..

[115] Lichtenberger M. (2004). Transfusion medicine in exotic pets. Clin. Tech. Small Anim. Pract..

[116] Lumeij J.T., Boschma Y., Mol J., de Kloet E.R., Van Den Brom W.E. (1987). Action of acth 1–24 upon plasma corticosterone concentrations in racing pigeons (*Columba livia domestica*). Avian Pathol..

[117] AMCRA Avis de l’AMCRA, Mesures Pour Une Utilisation Responsable des Antibiotiques Chez les Pigeons Voyaguers. https://www.amcra.be/fr/recent-nieuws/amcra-a-rdig-un-avis-pour-lapplication-de-mesures-favorisant-un-usage-responsable-des-antibiotiques-chez-les-pigeons-voyageurs/?lid=15618.

[118] Marlier D. Comparaison de l’antibiorésistance vis-à-vis de la fluméquine et de l’enrofloxacine chez des souches d’*Escherichia coli* d’oiseaux de compagnie ou d’oiseaux de production. Proceedings of the 3ème Colloque International de Bactériologie Francophone.

[119] Marlier D., Duchatel J.P., Vindevogel H. (1994). Quel antibiotique utiliser pour traiter les affections respiratoires antérieures chez le pigeon. Ann. Med. Vet..

[120] Kimpe A., Decemberostere A., Marchtel A., Haesebrouck F., Devriese L.A. (2002). Prevalence of antimicrobial resistance among pigeon isolates of *Streptococcus gallolyticus*, *Escherichia coli* and *Salmonella enterica* serotype Typhimurium. Avian Pathol..

